# Change in medical and health care decision-making patterns at the End-of-Life: A cohort of the very old people

**DOI:** 10.1093/geroni/igab046.2851

**Published:** 2021-12-17

**Authors:** Xinran Liu, Steven Albert

**Affiliations:** University of Pittsburgh, Pittsburgh, Pennsylvania, United States

## Abstract

How does medical and healthcare decision-making among the very old people change in their last year before death? We explored patterns of decision-making in the Health ABC cohort study in 2011-14 (years 15-17), which involved 12 waves of quarterly phone interviews. When the participant was unable to do the interview, a proxy completed it instead. We identified a sample of 291 decedents (aged 90.0±2.9 at death, 35.7% Black, 52.6% female) with at least 1-year follow-up before death. Percentages of decedents who have made medical or healthcare decisions in the last four quarters before death were 32.0%, 31.2%, 32.6%, 41.9%, respectively. Decedents made more healthcare decisions in the last quarter before death (P<0.01), compared to the baseline. Across all quarters, decision-making is most in need for medications (17.6%), hospital admission (13.2%), and ER/urgent care visit (13.2%). We matched a 1:1 sample of survivors at the time of death by race, sex, and age (within ±3 years). In random effects models with multiple imputations of missing data, we found that decedents were more likely to make healthcare decisions than survivors across all four quarters before death or censor (Odds ratio=1.99, 95%CI: 1.49-2.65, P<0.01). The significance still held after adjusting for age, female, race, education, and interview methods. Overall, compared to matched survivors, the frequency of making medical and healthcare decisions among the very old decedents has already been high in the last year before death. This frequency rose sharply in the last quarter before death.

